# Acetyltransferase p300 Is a Putative Epidrug Target for Amelioration of Cellular Aging-Related Cardiovascular Disease

**DOI:** 10.3390/cells10112839

**Published:** 2021-10-22

**Authors:** Asish K. Ghosh

**Affiliations:** Feinberg Cardiovascular and Renal Research Institute, Feinberg School of Medicine, Northwestern University, Chicago, IL 60611, USA; a-ghosh2@northwestern.edu

**Keywords:** acetyltransferase p300, small molecule inhibitors, cellular senescence, diabetes, TGF-β, vascular calcification, hypertension, heart disease

## Abstract

Cardiovascular disease is the leading cause of accelerated as well as chronological aging-related human morbidity and mortality worldwide. Genetic, immunologic, unhealthy lifestyles including daily consumption of high-carb/high-fat fast food, lack of exercise, drug addiction, cigarette smoke, alcoholism, and exposure to environmental pollutants like particulate matter (PM)-induced stresses contribute profoundly to accelerated and chronological cardiovascular aging and associated life threatening diseases. All these stressors alter gene expression epigenetically either through activation or repression of gene transcription via alteration of chromatin remodeling enzymes and chromatin landscape by DNA methylation or histone methylation or histone acetylation. Acetyltransferase p300, a major epigenetic writer of acetylation on histones and transcription factors, contributes significantly to modifications of chromatin landscape of genes involved in cellular aging and cardiovascular diseases. In this review, the key findings those implicate acetyltransferase p300 as a major contributor to cellular senescence or aging related cardiovascular pathologies including vascular dysfunction, cardiac hypertrophy, myocardial infarction, cardiac fibrosis, systolic/diastolic dysfunction, and aortic valve calcification are discussed. The efficacy of natural or synthetic small molecule inhibitor targeting acetyltransferase p300 in amelioration of stress-induced dysregulated gene expression, cellular aging, and cardiovascular disease in preclinical study is also discussed.

## 1. Introduction

Epigenetics plays a pivotal role in organismal gametogenesis, organogenesis, fetal growth, post-natal growth, adulthood physical/mental maturity, and aging [1]. Depending on the environment of individual’s habitat and lifestyle, epigenetic modifications of gene expression play crucial roles in health span, lifespan and aging related disease development including cardiovascular disease [1,2]. Epigenetic regulation of gene expression at the levels of chromatin organization and transcription are controlled by the balance of DNA methylation and DNA demethylation, histone acetylation and deacetylation, histone methylation and demethylation by epigenetic regulators or chromatin modifying enzymes. DNA methylation at CpG site is associated with transcriptional repression and regulated by epigenetic writers like DNA methyltransferases, epigenetic readers such as methyl CpG binding domain protein and transcription factors, and epigenetic eraser like DNA demethylase [3]. However, unlike DNA methylation, histone methylation is associated with both transcriptional activation as well as transcriptional repression depending on methylation of a specific histone lysine residue. For example, while H3K9me3 is associated with transcriptional repression; H3K4me1/me3 is associated with transcriptional activation. Histone methylation is regulated by epigenetic writers like histone methyltransferases, epigenetic readers such as tudor domain and mbt domain, and epigenetic erasers like histone demethylases [3]. In contrast, histone acetylation is associated with transcriptional activation and controlled by epigenetic writer histone and factor acetyltransferases like p300, epigenetic readers like brd/bet bromodomain, and epigenetic erasers such as zinc-dependent histone deacetylases or NAD-dependent sirtuins [4,5]. It is important to note that histone modifications in a gene is position specific and thus can distinguish different domains of a gene like promoter and enhancer during inactive, primed, and active chromatin states [6]. For instance, the histone acetylation and methylation at enhancer and promoter are distinct in a transcriptionally active gene. Similarly, the histone acetylation and methylation in the enhancer of a transcriptionally active gene are distinct from inactive and primed enhancer (reviewed in [6]). Epigenetic modification of gene expression also takes place at the post transcriptional level by non-coding RNAs including microRNAs, lncRNAs, and circRNAs that is associated with transcriptional repression [2]. Numerous studies have been conducted to define the roles of acetyltransferase p300 in cellular growth, differentiation, senescence, and disease development. In this article, the significant contribution of epigenetic regulator acetyltransferase p300 in cellular senescence/aging related cardiovascular disease is highlighted.

Acetyltransferase p300 plays significant roles in numerous cellular processes including proliferation, migration, differentiation, senescence, and apoptosis through chromatin remodeling in the regulatory regions of genes as an epigenetic regulator and/or an interacting coactivator with specific transcription factors of genes involved in those cellular processes [7,8,9]. For example, under normal growth conditions, fibroblasts synthesize and secrete low levels of extracellular matrix protein type I collagen using basal transcriptional machinery [10]. However, when fibroblasts are exposed to pleiotrophic cytokine transforming growth factor-beta (TGF-β), activated TGF-β receptor complex kinase phosphorylates cytoplasmic R-Smad2/3 that is followed by the interaction of pSmad2/3 with Co-Smad4 and translocation of Smad2/3-Smad4 heterodimers into the nucleus. Activated Smad2/3/4 complex binds to Smad-binding element of collagen promoter and interacts with and recruits acetyltransferase p300 to the transcriptional complex. Activated transcriptional complex significantly increases the transcription of collagen gene. Therefore, presence of acetyltransferase p300 or unphosphorylated Smad proteins in unstimulated cells are not sufficient to activate target gene expression, because signal specific modifications of p300 and its interacting factors are required for protein-protein interaction, nuclear translocation, and stimulation of target gene transcription [10,11,12,13]. The target gene specificity of acetyltransferase p300 is controlled by signal specific posttranslational modifications viz. phosphorylations by different kinases ([14] and references therein), autoacetylation [15], and methylation by coactivator-associated arginine methyltransferase 1 [16] in a cell type and context dependent manner. Abnormality in p300 acetyltransferase activity or other functional domains due to point or deletion mutations or non-physiological elevated levels of acetyltransferase activity, is associated with the development of numerous diseases including aging and cardiovascular diseases [9,17]. Therefore, acetyltransferase p300 controls a wide variety of pathological pathways as an epigenetic regulator of chromatin remodeling, and as a transcriptional coactivator of target gene expression. Here, the cellular and preclinical studies on the significance of acetyltransferase p300 in cellular aging and cardiovascular disease are discussed.

## 2. Acetyltransferase p300 in Cellular Senescence and Aging

The cellular senescence or irreversible cellular growth arrest contributes to stress-induced accelerated aging related pathologies [18]. Like genetic alteration-induced stresses, environmental stresses are also implicated in induction of cellular senescence, and, thus organismal aging processes. Environmental stress-induced alteration of chromatin-remodeling enzymes and other epigenetic regulators contribute significantly to the altered expression of genes involved in cellular senescence. Emerging evidence indicates that epigenetic regulator acetyltransferase p300 plays critical roles in cellular senescence and aging. Recently, Sen and colleagues [19] identified key epigenetic regulators involved in cellular replicative senescence. Using short hairpin RNAs (shRNA) for silencing numerous epigenetic regulators and high throughput screening, this study identified acetyltransferase p300, not related CBP, as a key epigenetic regulator that boosts the formation of super enhancer in the non-coding regulatory element of the genome that augments senescence-specific gene expression. This study further showed that shRNA-mediated depletion of the acetyltransferase p300 inhibits senescence-related gene expression and the cellular senescence process as evidenced by decreased telomere dysfunction-induced foci per cell, significantly less SA-β-Gal (senescent marker) positivity, and significantly higher 5-ethynyl-2′-deoxyuridine (EdU) positivity (proliferation marker) compared to control cells [19]. Based on these novel findings, authors concluded that acetyltransferase p300 may be a potential epidrug target for amelioration of stress-induced cellular senescence and aging related pathologies.

### 2.1. Cardiac Senescence

Numerous cellular, preclinical, and clinical studies have established that cardiovascular aging is largely characterized by stress-induced pathological cardiomyocyte hypertrophy, mitochondrial and metabolic dysfunction, cardiomyocyte senescence, cardiac fibroblasts and endothelial dysfunction, pathological matrix remodeling, decreased cardiac function, and ultimate cardiac arrest. Although, in adults, cardiomyocytes are terminally differentiated and non-proliferating, stress-induced cardiomyocytes show several traits of cellular senescence including DNA damage, endoplasmic reticulum stress, mitochondrial dysfunction, contractile dysfunction, hypertrophic growth, and senescence-associated secretory phenotype (SASP) [20,21,22]. The senescent cardiomyocyte-secreted SASP includes CCN1, IL1α, IL1β, and IL6, TNFα, MCP1, Edn3, TGFβ, and GDF15. While in healthy functional hearts, coordination of cardiomyocytes with cardiac fibroblasts, endothelial, and other cardiac cells is essential, cardiomyocyte senescence processes are also contributed to by stress-induced dysfunctional/or senescent fibroblasts and endothelial cells. [22,23,24]. Interestingly, an elevated level of acetyltransferase p300 has been implicated in doxorubicin-induced cardiac pathologies. Using young (2 months) and aged (10 months) SAMP8 mice (senescence accelerated mice prone 8, a murine model of accelerated aging), Sin and colleagues [25] reported that doxorubicin treatment significantly reduces the levels of SIRT1 deacetylases and increases the levels of acetyltransferase p300. Furthermore, the doxorubicin-induced imbalance between acetyltransferase p300 and deacetylase SIRT1 is associated with impaired cardiac function including fractional shortening and ejection fraction in both young and aged experimental groups. Importantly, resveratrol, a natural polyphenol, attenuates doxorubicin-induced reduction of SIRT1, induction of acetyltransferase p300 and impaired cardiac function indicating the significant role of epigenetic regulators acetyltransferase p300 and deacetylase SIRT1 in regulation of doxorubicin-induced cardiotoxicity and abnormal cardiac function [25]. Doxorubicin-induced accelerated cardiomyocyte senescence causes cardiotoxicity, cardiac tissue damage, and congestive heart failure [26,27]. Previously, we demonstrated that doxorubicin induces accelerated cellular senescence or aging processes in three cardiac cell types including cardiomyocytes, cardiac fibroblasts, and vascular endothelial cells as evidenced by elevated levels of senescence regulators p16, p21, p53, PAI-1, and senescence marker SA-β-Gal in doxorubicin treated cardiac cells [28]. Together, these results suggest that cardiac cellular senescence contributes to doxorubicin-induced cardiotoxicity and heart failure [25,26,27,28], and acetyltransferase p300 plays a significant role in cardiac cellular aging.

### 2.2. Diabetes-Induced Vascular Senescence and Dysfunction

Cellular senescence also plays pivotal roles in diabetes-induced vascular dysfunction and a wide variety of cardiovascular pathologies including hypertension, atherosclerosis, cardiac hypertrophy, pathological matrix remodeling, and heart failure. Importantly, diabetes-induced vascular dysfunction is epigenetically controlled by the fine balance of acetylation and deacetylation of transcription factors/regulators. Accumulated evidence reveals that acetyltransferase p300 plays a pivotal role in diabetes-induced accelerated cellular senescence and aging-related pathologies through alterations of different cellular programs [29,30,31]. It has been reported that the endothelial cells derived from gestational diabetes umbilical cord display accelerated senescence phenotype as evidenced by increased expression of senescence regulators including p16, p21, and p53 and senescence marker SA-β-Gal compared to control human umbilical vein endothelial cells (HUVECs). Interestingly, the levels of acetyltransferase p300 and acetylated p53 (K382) are significantly elevated in endothelial cells derived from a gestational diabetes umbilical cord compared to control, indicating the possible involvement of acetyltransferase p300 in diabetes-induced vascular endothelial senescence [29]. Furthermore, Mortuza and colleagues [30] demonstrated that vascular endothelial cells undergo senescence processes in response to high glucose, a pathological milieu of diabetic condition. Consistent with this observation, the glomerular and retinal blood vessels show increased cellular senescence as marked by elevated levels of SA-β-Gal positivity in diabetic mice compared to controls. The phenotypic changes of senescent endothelial cells under diabetic milieu are associated with downregulation of Sirtuins 1-7 (class III deacetylases) and upregulation of acetyltransferase p300. Similarly, the downregulation of SIRT1 activity and upregulation of acetyltransferase p300 are evident in renal tissues derived from streptozotocin-induced diabetic mice. This study identified the existence of a feedback loop in regulation of class III deacetylase Sirtuins and acetyltransferase p300 where Sirtuin silencing leads to augmentation of acetyltransferase p300 and p300 silencing blocks high glucose-induced suppression of Sirtuins in endothelial cells. Furthermore, downregulation of acetyltransferase p300 is linked with increased FOXO1 activity and induction of mitochondrial antioxidant MnSOD [30]. As acetyltransferase p300 plays a key role in high glucose-induced oxidative stress through suppression of antioxidant genes, pharmacological inhibition of acetyltransferase p300 activity may be an ideal approach to reduce diabetes-induced endothelial senescence and associated aging related cardiovascular pathologies in diabetic patients. Similarly, HUVECs exposed to high glucose induces endothelial senescence that is associated with elevated levels of reactive oxygen species (ROS), and acetyltransferase p300, and decreased levels of deacetylase SIRT1 [31]. Furthermore, acetyltransferase p300 acetylates cell cycle regulator p53 at K382 and increases its stability and transcriptional activity. Activated p53 binds to cell cycle inhibitor p21 gene promoter and activates its transcription. Thus, high glucose-induced acetyltransferase p300 contributes to endothelial senescence through acetylation/activation of senescence regulator p53 and its downstream target gene p21. This study further demonstrate that resveratrol or metformin, known repressor of p300 and activator of SIRT1, blocks high glucose-induced vascular senescence [31]. Therefore, these results clearly indicate the significance of acetyltransferase p300 in epigenetic regulation of endothelial senescence and vascular dysfunction.

### 2.3. Vascular Senescence in Atherosclerosis

Accelerated cellular senescence is also a major contributor in atherosclerosis or atherosclerotic plaque formation in aorta, a common vascular pathology during chronological aging as well as stress-induced accelerated vascular aging [32]. Vascular senescence in atherosclerosis is characterized by irreversible growth arrest or senescence, DNA damage, alteration in epigenetic footprint, and vascular cell dysfunction [32]. The contribution of reactive oxygen species (ROS) in atherosclerotic plaque formation and underlying molecular basis has been delineated by Vlad and colleagues [33]. This study demonstrated that macrophages expressed NADPH oxidase 5 (Nox5) contributes to the generation of ROS and atherosclerotic plaque formation. Interestingly, the protein levels of acetyltransferase p300, Ac-H3K27, and NADPH oxidase 5 (Nox5) are significantly elevated in human atherosclerotic samples and colocalized in the macrophage rich area within carotid atherosclerotic plaques. Treatment of macrophages with lipopolysaccharide (LPS) induces the levels of Nox5 through increased recruitment of acetyltransferase p300 to the Nox5 gene promoter and histone acetylation. Furthermore, while overexpressed p300 enhances the Nox5 gene expression in macrophages, inhibition of acetyltransferase p300 with small molecule C646 blocks LPS-induced Nox5 gene expression in macrophages indicating the significance of acetyltransferase p300 in epigenetic regulation of Nox5, a major contributor to oxidative stress during atherosclerotic plaque development [33]. Additionally, Shah and colleagues [34] reported that acetyltransferase p300 controls vascular senescence in atherosclerosis where stress-induced ROS augments DNA damage that leads to accumulation of different modified DNA bases like 8-oxo-7,8 dihydroguanine (8oxoG). 8oxoG is removed or repaired by enzyme OGG1 glycosylase in vascular smooth muscle cells (VSMCs). Acetylation of OGG1 is critical for its repair function. Interestingly, while acetyltransferase p300 acetylates OGG1, class III deacetylase SIRT1 deacetylates OGG1. In atherosclerotic plaque VSMCs, the 8oxoG removal by OGG1 is impaired [34]. Mechanistically, reduced levels of acetyltransferase p300 in VSMCs from atherosclerotic plaque may cause impaired acetylation of OGG1 that disrupts its activity and increases the levels of 8oxoG. The significance of OGG1 acetylation and its contribution in controlling atherosclerosis has been further investigated in murine models where control ApoE^−/−^, OGG1^−/−^ApoE^−/−^, SM22α-OGG1 ApoE^−/−^, and SM22α-OGG1^K-R^ ApoE^–/–^ mice were fed a high-fat diet for 14 weeks. While OGG1^−/−^ApoE^−/−^ mice display elevated 8oxoG and increased atherosclerotic plaque compared to control ApoE^–/–^ mice, VSMC specific OGG1 overexpressing SM22α-OGG1 ApoE^−^^/^^−^ mice are protected from high-fat-diet-induced atherosclerosis. Interestingly, the protection from atherosclerosis is diminished in acetylation mutant SM22α-OGG1^K-R^ ApoE^−^^/^^−^mice. Together, the results of this study implicate acetyltransferase p300 and deacetylase SIRT1-mediated regulation of OGG1 in controlling atherosclerosis through regulation of oxidative stress-induced cellular senescence [34]. Collectively, these studies further implicate the physiological level of acetyltransferase p300 in amelioration of vascular senescence and atherosclerosis.

### 2.4. Possible Underlying Mechanisms and Epidrug Development

**Figure 1:**
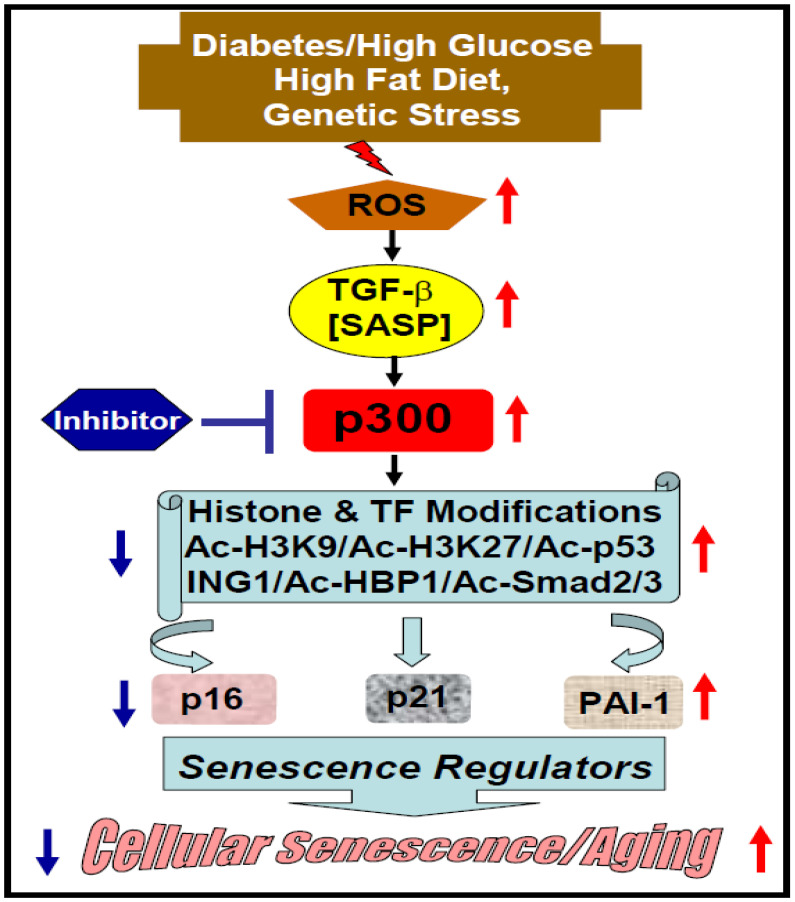
Model depicting the possible contribution of acetyltransferase p300 in cellular senescence/aging. ROS: Reactive Oxygen Species, TGF-β: Transforming Growth Factor-β. TF: Transcription factor, SASP: Senescence-Associated Secretory Phenotype, Ac: Acetylated histone/TF, ING1: Inhibitor of Growth 1.

The obvious question is how acetyltransferase p300 controls cellular senescence. Is it through epigenetic regulation of key senescence regulators? Li and colleagues [35] reported that the inhibitor of growth (ING) family proteins ING1 isoforms ING1a and ING1b may play a role in replicative cellular senescence. ING1b induces the expression levels of p16, a key marker and regulator of cellular senescence, and accelerates fibroblast senescence. Interestingly, ING1b interacts with acetyltransferase p300 and recruits it to p16 promoter that is required for the increased expression of p16. Importantly, acetyltransferase p300 inhibitor curcumin blocks p16 expression and cellular senescence [35]. Mechanistically, ING1 acts as a reader of H3K4me3 histone marks (associated with transcriptional activator) and recruits acetyltransferase or HDAC for alteration of chromatin architecture—as relaxed (transcriptional activation) or as condensed (transcriptional repression), respectively [36]. In addition, the expression of p16 is regulated by transcription factor HBP1, a high mobility group box containing protein1. HBP1 binds to a specific sequence (GGGGGTAGGGGG) in the p16 promoter and stimulates its transcription. Importantly, HBP1 interacts with acetyltransferase p300 and this interaction is required for transactivation of p16 in fibroblasts. Mechanistically, recruitment of acetyltransferase p300 by HBP1 is associated with HBP1 acetylation at K419, which is essential for increased expression of p16 and accelerated premature fibroblast senescence [37]. These results implicate acetyltransferase p300 in epigenetic regulation of cellular senescence through activation of senescence regulator p16. However, p300 mediated stimulation of p16 may also be achieved by acetylation of histone in the p16 gene promoter. Like senescence regulator p16, acetyltransferase p300 also controls other key senescence regulators p21 and PAI-1 [38]. In response to TGF-β, a SASP, elevated acetyltransferase p300 increases the acetylation of H3K9, H3K14 and receptor Smad, Smad2/3 on p21 promoter and its expression in rat mesangial cells [38]. Thus, acetyltransferase p300 may play a key role in cellular senescence through induction of cell cycle and senescence regulator p21. This is consistent with the later observation that elevated acetyltransferase p300 stimulates the expression of senescence regulator p21 and cellular senescence in a diabetic milieu [31]. Similarly, Plasminogen activator inhibitor-1 (PAI-1), a key regulator of cellular senescence and aging related pathologies [18], is also regulated by acetyltransferase p300. In response to TGF-β signaling, activated Smad binds to Smad binding element on PAI-1 promoter, recruits p300 and interacts with Sp1 where acetyltransferase p300 activates PAI-1 gene expression via increased acetylation of H3K9/H3K14 and Smads [38]. Together, these results from different experimental setups clearly indicate that cellular or vascular senescence is tightly controlled epigenetically by acetyltransferase p300 (Figure 1). Although, based on different senescence stressors and pathological milieus, the alteration levels of acetyltransferase p300 activity are different, but it is reasonable to conclude that any significant alteration in acetyltransferase p300 activity contributes to cellular aging pathologies, and thus physiological level of this epigenetic writer is essential to maintaining cellular homeostasis under different pathological stresses. Therefore, acetyltransferase p300 may be an ideal therapeutic target for amelioration of cellular senescence and aging associated cardiovascular pathologies including diabetes-induced vascular dysfunction, anticancer drug doxorubicin-induced cardiotoxicity, and high-fat-diet-induced atherosclerosis.

Recently, the efficacy of CCS1477, a novel small-molecule inhibitor that selectively binds to the p300/CBP bromodomain, has been tested in amelioration of prostate cancer [39]. The small molecule CCS1477 inhibits proliferation in prostate cancer cell lines. Importantly, early clinical studies using patient-derived mouse xenograft suggest that CCS1477 is a promising epidrug for the treatment of prostate cancer [39]. Therefore, based on future compelling evidence on the efficacy and safety, repurposing of such drug-like small molecule inhibitor targeting p300 for the treatment of cardiovascular disease will be an attractive approach.

## 3. Acetyltransferase p300 Boosts Cardiac Hypertrophy, Myocardial Fibrosis, and Heart Failure

Sustained high blood pressure or left ventricular pressure overload due to blockage in the aorta/aortic branch leads to pathological cardiac hypertrophy, matrix remodeling, and heart failure [40,41]. As mentioned earlier, cardiovascular pathologies such as cardiac hypertrophy and associated myocardial matrix remodeling are epigenetically regulated where acetyltransferase p300 plays a pivotal role in disease development and progression. Acetyltransferase p300 controls the expression levels of target genes involved in cardiac hypertrophy and fibrogenesis at the multiple levels. First, as a chromatin remodeling enzyme, it acetylates the specific histone lysine residues at the target gene regulatory region that is favorable for transcriptional activation; second, as an epigenetic regulator, it also acetylates and modulates transcription factors; and third, as a transcriptional coactivator, it forms a physical/functional bridge between basal transcriptional initiation complex and enhancer binding transcription factors. In this section, the key roles of acetyltransferase p300, a chromatin remodeling enzymes, an epigenetic regulator, and a transcriptional coactivator, in regulation of cardiac hypertrophic and fibrogenic genes in response to different stressors and its impact in cardiovascular disease development are discussed.

### 3.1. α-Adrenergic Receptor Agonist Phenylephrine-Induced Cardiac Hypertrophy and Fibrosis: Epigenetic Regulation by Acetyltransferase p300

Phenylephrine (PE)-induced cardiac hypertrophy and myocardial fibrosis in rodents is used as a widely-accepted animal model of hypertension induced cardiovascular complications in humans. Plenylephrine is an α-adrenergic receptor agonist and a vasoconstrictor that increases blood pressure and decreases heart rate. Interestingly, acetyltransferase p300 plays a key role in PE-induced cardiovascular pathologies. PE induces the acetylation of zinc finger protein GATA-4 (GATA binding protein 4), a major transcription factor involved in cardiomyocyte hypertrophy. Interestingly, a dominant negative mutant form of p300 blocks PE-induced GATA4 acetylation and cardiac hypertrophic responses in neonatal rat ventricular cardiomyocytes. Further, α-MHC promoter driven cardiac specific overexpression of p300 leads to increased acetylation of GATA4 that is associated with elevated expression of hypertrophy responsive genes including β-MHC, ANP and ET-1, and left ventricular dysfunction [42]. Collectively, these results indicate that acetyltransferase p300-induced acetylation of transcription factors including GATA4 plays a pivotal role in PE-induced cardiac hypertrophy and heart failure.

Later, Sunagawa and colleagues [43] identified Cdk9 as an interacting factor with p300-GATA4 complex, and Cdk9 kinase activity is required for PE-induced phosphorylation of p300. Acetyltransferase p300 is essential for increased acetylation of GATA4 and GATA4-Cdk9 complex formation. Furthermore, the recruitment of Cdk/p300/GATA4 complex on the hypertrophy responsive genes enhances their expression, and thus cardiomyocyte hypertrophy [43]. Therefore, neutralization of stress-induced increased p300 acetyltransferase activity is an ideal approach to ameliorate cardiac hypertrophy and heart failure. In contrast to Cdk9, a receptor for activated protein kinaseC1 (RACK1), a novel GATA4 interacting protein, acts as a repressor of PE-induced cardiomyocyte hypertrophy and hypertrophic gene expression including ANP, BNP, and ET-1. Overexpressed RACK1 interacts with GATA4 and disrupts PE-induced p300-GATA4 activation complex formation on hypertrophic genes and thus suppresses hypertrophy in neonatal rat cardiomyocytes and cardiac hypertrophy in salt-sensitive Dahl (DS) rat model of hypertension. These results further indicate the significant contribution of acetyltransferase p300 in cardiac hypertrophy and heart failure [44]. Interestingly, a recent study showed that while the levels of Ac-H3K9 on ANP, BNP, and β-MHC promoters are significantly increased during left ventricular hypertrophy stage in Dahl salt-sensitive rats after 6 weeks salt exposure, the levels of Ac-H3K122 on ANP, BNP and β-MHC promoters are elevated at heart failure stage after 15 weeks salt exposure compared to age matched salt-resistant rats [45]. These results clearly established the stage specific recruitment of acetyltransferase p300 to target genes and lysine acetylation of specific histones to target gene promoters play critical role in progression of cardiac pathologies. Authors further delineated the underlying mechanism of stage-specific recruitment of p300 to hypertrophy responsive gene promoters and showed that the interaction of acetyltransferase p300 with BRG1, a functional subunit of chromatin remodeling complex, and their recruitments to target genes are increased in late heart failure stage but not in early left ventricular hypertrophy stage [45]. Together, these results indicate that p300-mediated epigenetic regulation of genes involved in hypertrophy and eventual heart failure are progressive and disease stage-specific. This study further established the specificity of acetyltransferase p300 activity in terms of acetylation of specific lysine residues of histones in a context-dependent manner.

As mentioned earlier, numerous studies implicate Sirtuin family members in the protection of cardiovascular pathologies [46] possibly through controlling acetyltransferase activity and the levels of histone acetylation. For example, Sirtuin-6 (SIRT6) is involved in cardioprotection including cardiac hypertrophy and heart failure. The levels of SIRT6 is significantly decreased in PE-induced rat neonatal cardiomyocytes and that is associated with elevated levels of acetyltransferase p300 and cardiomyocyte hypertrophy. Overexpressed SIRT6 attenuates PE-induced cardiac hypertrophy through reduction of p300 by proteosomal degradation. Further, SiRNA-mediated downregulation of p300 leads to abrogation of PE-induced cardiomyocyte hypertrophy strongly indicate the significance of p300 as a prohypertrophic candidate and SIRT-6 exerts its anti-hypertrophic action through downregulation of key prohypertrophic factor acetyltransferase p300 [47]. The essential roles of p300 and associated factor pCAF in PE-induced cardiac hypertrophy has been further recognized by the observation that anacardic acid, an alkyl salicylic acid and inhibitor of acetyltransferases, blocks PE-induced cardiac hypertrophy through inhibition of acetylation of histone H3K9 and myocyte enhancer factor-2 (Mef-2) DNA binding activity. Thus, inhibition of acetyltransferase activity normalize hypertrophic gene expression including ANP, BNP, Cx43, cTnT, and β-MHC and mitigate murine hypertrophic cardiomyopathy [48]. A recent study demonstrates that while overexpression of p300 in neonatal rat cardiomyocytes leads to increased acetylation of H3K9 and H3K122 and increased expression of ANP, BNP, and β-MHC, siRNA-mediated depletion of p300 in cardiomyocytes blunts PE-induced acetylation of H3K9 and H3K122, and the expression of PE-induced hypertrophy responsive genes [45]. These findings further signify the key role of acetyltransferase p300 in specific histone lysine acetylation and PE-induced pathological cardiac hypertrophy. Therefore, it is quite reasonable to predict that acetyltransferase p300 is a potent target of epidrug development for the therapy of cardiac disease.

### 3.2. Peptide Hormone Angiotensin II-Induced Cardiac Hypertrophy and Fibrosis: Pivotal Role of Epigenetic Regulator Acetyltransferase p300

Acetyltransferase p300 plays a pivotal role in AngiotensinII-induced cardiac hypertrophy and myocardial fibrogenesis. Angiotensin II (AngII) is an 8-amino acids peptide (Asp-Arg-Val-Tyr-Ile-His-Pro-Phe) hormone produced from the precursor angiotensinogen (485 amino acids peptide, secreted from liver) through cleavage by renin (produced in kidney) to Angiotensin I (10 amino acids peptide). Angiotensin converting enzyme (ACE) (produced in lungs and kidney) convert Angiotensin I to AngII (produced in cardiovascular tissues). AngII, a known vasoconstrictor that signals through AngII type I (AT1) and AngII type II (AT2) receptors, increases blood pressure and involved in inflammation, cardiac hypertrophy, fibrogenesis, kidney disease, muscle atrophy, oxidative stress, mitochondrial dysfunction, and aging. (Reviewed in [49]). AngII peptide is widely used as a vasoconstrictor in hypertension related preclinical cardiovascular and renal disease research. We have demonstrated the pivotal roles of acetyltransferase p300 in AngII-mediated hypertension-induced cardiac hypertrophy and myocardial fibrogenesis. AngII infusion in mice induces the level of acetyltransferase p300 in myocardial tissues. Pharmacological inhibition of p300 acetyltransferase activity with L002, a small molecule inhibitor, inhibits AngII-mediated hypertension-induced ventricular wall thickness, cardiac hypertrophy, and fibrosis without mitigation of AngII-induced increased blood pressure. Mechanistically, p300 inhibitor L002 ameliorates AngII downstream effector TGF-β-induced cardiac abnormalities through inhibition of specific histone acetylation, myofibroblast differentiation, and matrix protein collagen synthesis in cardiac fibroblasts [13]. Interestingly, pharmacological inhibition of acetyltransferase activity of p300 is associated with abrogation of TGF-β-induced increased expression of AT1 receptor (activator of profibrogenic signaling) and TGF-β-induced suppression of AT2 receptor (repressor of profibrogenic signaling) in cardiac fibroblasts [13] indicating the beneficial effect of p300 acetyltransferase activity neutralization. Furthermore, p300 inhibitors L002 and C646 reverse AngII-mediated hypertension-induced histone H3K9 acetylation, myofibroblast differentiation in myocardial tissues, left ventricular wall thickness, cardiac hypertrophy and myocardial fibrosis in a murine model [50]. Together, these results clearly established the significance of epigenetic regulator acetyltransferase p300 as a potential target of epidrug development for amelioration of hypertension-induced pathological cardiac hypertrophy and myocardial fibrosis, the leading cause of heart-failure-related deaths.

### 3.3. Acetyltransferase p300 Contributes to Transverse Aortic Constriction-Induced Cardiac Hypertrophy and Fibrosis

The significant involvement of acetyltransferase p300 in transverse aortic constriction (TAC)-mediated left ventricular pressure overload-induced cardiovascular pathologies is well documented. In response to hypertrophic signals, the transcriptional activity of non-DNA binding myocardin, a coactivator of serum response factor (SRF), is increased, which leads to increased expression levels of its target genes. The expression levels of myocardin is significantly elevated in left ventricular pressure overload-induced murine heart that is associated with cardiac hypertrophy as evidenced by elevated expressions of hypertrophic markers ANP and β-MHC [51]. Furthermore, overexpressed myocardin induces hypertrophy responsive gene expression and hypertrophy in neonatal rat cardiomyocyte. Most importantly, acetyltransferase p300 is required for myocardin regulated hypertrophic gene expression, where myocardin interacts with p300, and this physical interaction augments myocardin transcriptional activity, hypertrophic gene expression, and cardiomyocyte hypertrophy [51]. Interestingly, acetylation of myocardin by acetyltransferase p300 enhances its stable complex formation with SRF and decreases its interaction with transcriptional repressor HDAC5 [52]. Furthermore, overexpressed dominant negative mutant myocardin (Myocd-K4R), deficient for acetylation, fails to stimulate expression of smooth muscle genes including SM22, SM-α-actin, and SM-MHC in fibroblasts indicating acetylation of myocardin by acetyltransferase p300 is essential for its transcriptional activation [52]. Similarly, overexpressed dominant negative mutant myocardin that lacks the p300 interacting domain, fails to stimulate expression of cardiac hypertrophy responsive genes α-actinin and ANP, and thus prevents PE-induced cardiomyocyte hypertrophy [51]. Together, these findings solidify the pivotal role of acetyltransferase p300 in cardiomyocyte hypertrophy in vitro and left ventricular pressure overload-induced cardiac hypertrophy and associated pathologies.

The significant role of p300-myocardin complex in left ventricular pressure overload-induced cardiac pathologies has been further documented by another study on the involvement of interferon regulatory factor 9 (IRF9) in cardiac pathologies. This study [53] identified IRF9 as a negative regulator of left ventricular pressure overload-induced cardiac hypertrophy and showed that IRF9 level is significantly elevated in TAC-induced murine hearts. In contrast to wildtype IRF9^+/+^mice, the IRF9^−^^/^^−^mice show worst cardiac hypertrophic and profibrogenic responses as evidenced by significantly elevated levels of ANP and β-MHC, and myocardial fibrosis. Furthermore, transgenic mice overexpressing IRF9 are protected from left ventricular pressure overload-induced pathological cardiac hypertrophy. Mechanistically, IRF9 interacts with myocardin that leads to dissociation of acetyltransferase p300 from functional myocardin-p300 complex and reduced transcriptional activity of myocardin in cultured cardiomyoblasts and neonatal rat cardiomyocytes [53]. Collectively, these results clearly demonstrate the involvement of acetyltransferase p300 in positive regulator myocardin- and negative regulator IRF9-modulated cardiac hypertrophy in response to ventricular pressure overload. Therefore, based on cellular and animal studies, it is reasonable to speculate that acetyltransferase p300 is an ideal druggable target for epitherapy of cardiac hypertrophy and associated pathologies. 

The involvement of acetyltransferase p300 in TAC-induced cardiovascular pathology and its potential as a druggable target has been further evidenced by the observation that acetyltransferase inhibitor anacardic acid, a plant product, effectively inhibits left ventricular pressure overload-induced acetyltransferase p300, p300/CBP associated factor (pCAF) and histone H3K9 acetylation in murine myocardial tissues [54]. Importantly, p300/pCAF inhibition by anacardic acid is associated with suppression of TAC-induced cardiac hypertrophy as evidenced by decreased heart weight and cardiomyocyte diameter. The inhibition of acetyltransferase p300 significantly ameliorates TAC-induced cardiac structural and functional abnormalities including left ventricular anterior wall thickness, interventricular septum, left ventricular end diastolic dimension, and left ventricular end systolic dimension. Mechanistically, acetyltransferase p300 inhibition is associated with decreased transcriptional activity of MEF2A and reduced expression of ANP and β-MHC compared to controls further indicating the potentiality of acetyltransferase inhibitor as an epidrug for the therapy of pressure overload-induced cardiac hypertrophy and heart failure [54]. Similar to anacardic acid, low dose of GO-Y030, a synthetic analogue of curcumin and highly effective inhibitor of acetyltransferase p300, blunts TAC-induced increased acetyltransferase p300 activity, H3K9 acetylation, cardiac hypertrophy, myocardial fibrosis, and systolic dysfunction in mice [55]. The results from these preclinical studies further recognized the pivotal role of elevated levels of acetyltransferase p300 in cardiac pathologies and its potentiality as a druggable target for epitherapy of cardiovascular disease.

A recent report suggests that the nucleotide-binding oligomerization domain 1 (Nod 1) and receptor-interacting protein 2 (RIP2) axis, involved in immune signaling and immunity [56], plays a significant role in left ventricular pressure overload-induced cardiac hypertrophy and matrix remodeling [57]. Interestingly, acetyltransferase p300 also plays a significant role in this cardiac pathological pathway. This study showed that the levels of Nod1 and RIP2 are significantly elevated in human failing hearts and post-TAC murine hearts. Both Nod1 deficient and RIP2 deficient mice are protected from TAC-mediated left ventricular pressure overload-induced cardiac hypertrophy and cardiac dysfunction as evidenced by decreased heart weight and hypertrophic marker genes ANP and BNP, decreased perivascular and interstitial fibrosis, improved left ventricle end-diastolic dimension and fractional shortening compared to that of post-TAC wildtype mice. Interestingly, the protection of Nod1 deficient or RIP2 deficient mice from TAC-induced pathologies are linked with significant reduction of acetyltransferase p300 activity and its interaction with key hypertrophic transcription factor GATA4, compared to elevated levels of p300 in post TAC wildtype mice [57]. These findings further implicated acetyltransferase p300 as a major contributor in left ventricular pressure overload-induced cardiac pathologies. Thus, acetyltransferase p300 is an ideal target of epidrug development for the therapy of left ventricular pressure overload-induced cardiac pathologies.

### 3.4. Genetic Models of Cardiac Hypertrophy, Fibrosis and Heart Failure: Contribution of Acetyltransferase p300

Acetyltransferase p300 also plays a pivotal role in cardiac pathologies in spontaneously hypertensive rats. The inbred spontaneously hypertensive rat (SHR), originated from Wister Kyoto rat (WKY) breeding, is used as a genetic animal model of high blood pressure or hypertension [58] and characterized by cardiac hypertrophy, increased hypertrophic gene expression, and apoptosis in myocardial tissues. Jin and colleagues [59] reported that both systolic and diastolic blood pressures in SHR are significantly higher compared to WKY rats. Increased blood pressure is associated with left ventricular hypertrophy and myocardial apoptosis. Importantly, the expression levels of p300 mRNA is significantly higher in SHR compared to WKY. This study further demonstrated that Gallic acid, a trihydroxybenzoic acid, efficiently blocks hypertension-induced cardiac hypertrophy in SHR rats through downregulation of acetyltransferase p300 expression along with inflammatory and apoptotic markers [59]. As Gallic acid is an inhibitor of acetyltransferases [60,61] including p300, and Gallic acid blunts increased expression of p300 in SHR [59], it is reasonable to interpret that acetyltransferase p300 is one of the key players contributing to cardiac pathologies in this genetic rodent model of hypertension and chronic heart failure. Acetyltransferase p300 is also involved in high-salt-diet-induced worst cardiac hypertrophy, myocardial fibrosis, and abnormal heart function in polycystic kidney disease 2-like 1 (PKD2L1)-deficient mice [62]. PKD2L1 protein is a multiple transmembrane domains containing calcium-regulated nonselective cation channel. PKD2L1 deficiency induces p300 acetyltransferase activity, and acetylation of H3K27 on the sodium/calcium exchange 1 (NCX1) promoter through repression of AMPK activity that causes increased NCX1 expression. Importantly, p300 acetyltransferase inhibitor curcumin reduces the PKD2L1 knockdown-induced increased levels of acetylated-H3K27 on NCX1 gene promoter and its expression in H9c2 cardiomyoblasts [62]. Together, these results support the notion that acetyltransferase p300 is a prominent contributor to cardiac pathologies.

Interestingly, acetyltransferase p300 also plays a critical role in gestational hypertension-induced cardiac hypertrophy in adult offspring [63]. Using female mice lacking anti-hypertrophic atrial natriuretic peptide (ANP^–/–^) gene as a genetic model of gestational hypertension, Armstrong and colleagues demonstrated that 10 weeks old ANP^+/–^(KO) offspring (derived from hypertensive ANP^–/–^ females and ANP^+/+^ males) develop significant cardiac hypertrophic phenotypes compared to age matched ANP^+/–^(WT) offspring (derived from normotensive ANP^+/+^ females and ANP^–/–^ males). In ANP^+/–^(KO) offspring, the significantly elevated levels of acetyltransferase p300 and acetylated GATA4/GATA6 contribute to gestational hypertension-induced cardiac hypertrophy [63]. Therefore, acetyltransferase p300 plays a key role in the development of cardiovascular pathologies including cardiac hypertrophy, fibrogenesis and diastolic dysfunction in response to gestational genetic stress. Like anti-hypertrophic and anti-fibrotic ANP deficiency-induced cardiac hypertrophy, acetyltransferase p300 also contributes to Klf15-deficiency-induced cardiovascular complications [64]. The Klf15, a zinc finger transcription factor, acts as a repressor of prohypertrophic responses and its expression is significantly lower in AngII-treated cardiomyocytes, murine myocardial tissues, and aorta. In response to AngII infusion, Klf15 deficient mice develop a severe aortapathy characterized by elastolysis, increased apoptosis and reduced medial thickness, and cardiomyopathy characterized by left ventricular dysfunction compared to AngII-induced wildtype mice indicating Klf15 deficient mice are more susceptible to develop stress-induced cardiovascular pathologies. Interestingly, acetyltransferase p300 is involved in Klf15 deficiency-induced cardiomyopathy and aortapathy as evidenced by the observation that acetyltransferase p300 inhibitor curcumin significantly ameliorates AngII-induced aortapathy and cardiomyopathy-related pathologies in Klf15 knockout mice [64]. Together, these observations further indicate that acetyltransferase p300 plays a major role in Klf15-deficiency-induced heart failure and aortic aneurism, and thus pharmacological inhibition of acetyltransferase p300 is an ideal approach to ameliorate genetic stress-induced cardiovascular pathologies. The significant role of acetyltransferase p300 in SIRT3-deficiency-induced decreased coronary flow reserve (CFR) and diastolic dysfunction has also been documented [65]. Like other Sirtuin family members, SIRT3 also shows cardioprotective features. A complete deficiency of SIRT3 is associated with impaired coronary flow reserve (CFR) as an indicator of obstructive coronary artery disease and microvascular dysfunction. Interestingly, the levels of acetyltransferase p300 and acetylated-H3K56 are significantly elevated in SIRT3 deficient myocardial tissues. Elevated acetyltransferase p300 significantly contributes to SIRT3-deficiency-induced CFR as p300 acetyltransferase specific inhibitor C646 treated SIRT3 deficient mice show improved CFR, ejection fraction, fractional shortening, reduced cardiac hypertrophy, and fibrosis. Furthermore, inhibition of p300 acetyltransferase activity with C646 significantly reduces the SIRT3 deficiency-induced elevated levels of inflammatory factors NF-kB and VCAM1 in myocardial tissues, increases the levels of eNOS and ameliorates endothelial dysfunction [65]. Therefore, acetyltransferase p300 plays a pivotal role in SIRT3 deficiency-induced endothelial dysfunction, CFR, cardiac hypertrophy and matrix remodeling. Although, the underlying molecular bases of cardiovascular disease development in different genetic models are different, but acetyltransferase p300 is a common and key contributor to cardiovascular pathologies in these different genetic models of cardiovascular disease. Thus, acetyltransferase p300 is a potent epidrug target for therapy of different stress-induced cardiovascular pathologies.

### 3.5. Involvement of p300 in Environmental Air Pollution and Alcohol-Induced Cardiovascular Disease

According to World Health Organization (WHO) estimation, ambient air pollution causes world-wide 4.2 million deaths per year due to cardiopulmonary and vascular disease including hypertension, pathological cardiac hypertrophy, heart failure, stroke, chronic obstructive pulmonary disease (COPD) and lung cancer. Several recent studies suggest that epigenetics plays a pivotal role in air-pollutant-induced pathologies [66,67,68]. Importantly, involvement of acetyltransferase p300 in air pollution, particulate matter (PM_2.5_)-induced cardiac hypertrophy and cardiovascular pathologies has also been reported. Wu and colleagues [69] demonstrated that in utero exposure to PM_2.5_ leads to low birth weight up to age 12 weeks. Interestingly, in utero PM_2.5_ exposed adult mice developed cardiac hypertrophy, and that is associated with increased expression of p300/CBP and increased acetylation of histone H3K9 and expression of GATA4 and Mef2e cardiac transcription factors. The increased expression of GATA4 and Mef2c in PM_2.5_ exposed mice are due to increased recruitment of p300/CBP to these gene promoters and acetylation of histone H3K9. These results further indicate that acetyltransferase p300 plays a pivotal role in pathological cardiac hypertrophy and remodeling in adult mice those exposed to PM_2.5_ in utero [69]. Similarly, a recent study also showed that PM_2.5_ exposure in utero is associated with cardiac injury related phenotypes including cardiac inflammation, interstitial edema and hypertrophy in adult offspring [70]. However, unlike previous reports [69], this study showed that PM_2.5_ exposure in utero causes downregulation of acetyltransferase p300 activity especially in male offspring, and that is associated with decreased recruitment of acetylated H3K9 to GATA4 promoter and its transcriptional repression. As GATA4 is a key transcription factor for embryonic heart development, authors suggested that downregulation as well as decreased acetylation of GATA4 may contribute to PM_2.5_-induced in utero heart injury and cardiac dysfunction in adult offspring [70]. However, the reason for the discrepancy between the results of these two studies is unclear.

Similar to air pollution exposure, exposure of wildtype pregnant mice to alcohol also leads to cardiac hypertrophy in fetal mice. Interestingly, ethanol exposure significantly induces the levels of histone acetyltransferase activity in fetal hearts. While increased recruitment of acetyltransferase including p300/CBP and pCAF to GATA4 and NKX2.5 promoters is associated with increased level of H3K9 acetylation and hypertrophic gene expression, inhibition of p300/pCAF with anacardic acid leads to attenuation of alcohol-induced recruitment of p300/pCAF to hypertrophy responsive gene promoters, decreased H3K9 acetylation, decreased expression of hypertrophy responsive genes and amelioration of cardiac hypertrophy [71,72]. These results further indicate the significance of acetyltransferase p300 in alcohol-induced cardiac hypertrophy and as a potent target for epitherapy of environmental pollutant- and alcohol stress-induced cardiovascular pathologies.

### 3.6. Myocardial Infarction Associated Cardiovascular Pathologies Are Epigenetically Modulated by Acetyltransferase p300

**Figure 2:**
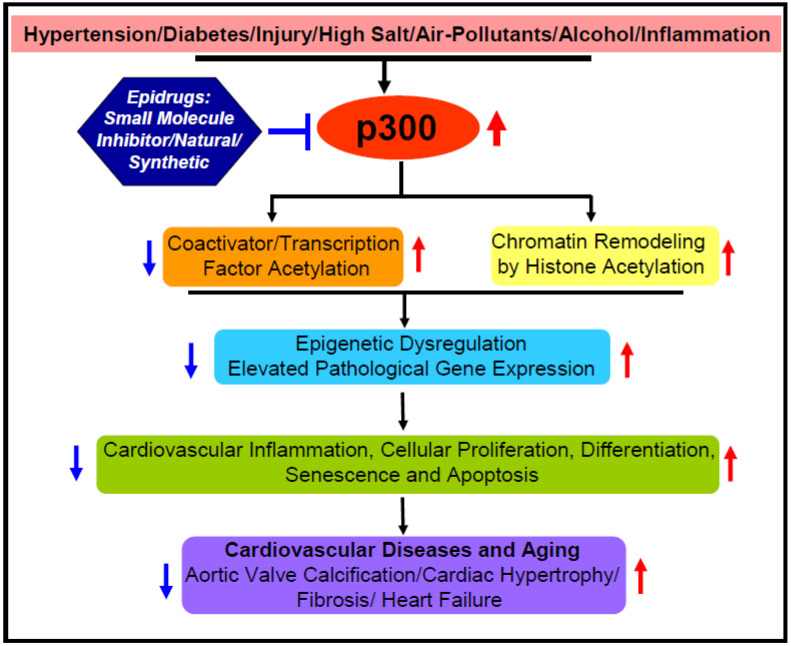
Model depicting the possible contribution of acetyltransferase p300 in cardiovascular disease.

Acetyltransferase p300 plays a key role in myocardial infarction or heart attack-induced cardiovascular pathologies. The essential role of acetyltransferase activity of p300 in matrix remodeling in left anterior descending coronary artery ligation-induced myocardial infarcted heart is documented by the observation that a p300 mutant transgenic mice, with 2-amino acid alteration in the acetyltransferase domain, are protected from myocardial infarction-induced left ventricular dilation, reduced systolic blood pressure and ventricular fibrosis. In contrast, wildtype acetyltransferase p300 expressing transgenic mice with myocardial infarction show significantly higher ventricular dilation and matrix remodeling compared to wildtype control mice. Furthermore, overexpressed wildtype acetyltransferase p300 in myocardial infarcted hearts increases GATA4 acetylation that contributes to worst cardiac pathologies in this murine model of heart failure [73]. Furthermore, acetyltransferase p300 inhibitor curcumin ameliorates the proximal left anterior descending coronary artery ligation-induced myocardial infarction-associated pathologies in rats including decreased left ventricular fractional shortening [74]. Using a novel and efficient drug delivery system, delivery of curcumin at 7 week post-myocardial infarcted murine hearts significantly reverses cardiomyocyte hypertrophy and perivascular fibrosis indicating the potentiality of acetyltransferase p300 as an epidrug target for therapy of heart-attack-induced established cardiac dysfunction [75]. The contribution of p300 in myocardial infarction-induced cardiac pathologies has been further evidenced in C1q/tumor necrosis factor related protein (CTRP3) overexpressed myocardial infarcted hearts. The level of CTRP3, an adipokine involved in multiple cellular processes including cardiovascular pathophysiology [76,77], is significantly decreased in left anterior descending coronary artery-induced myocardial infarcted rat hearts. Adeno-overexpressed CTRP3 alleviates myocardial infarction-induced cardiac hypertrophy, cardiac fibrosis and left ventricular end-diastolic pressure. CTRP3 exerts its anti-hypertrophic/anti-fibrotic effect through inhibition of Smad3 activation and Smad3 interaction with acetyltransferase p300 [78], an essential factor for fibroblast to myofibroblast differentiation and matrix protein collagen synthesis, the major contributor to fibrosis [13,50,78]. Collectively, these results further signify the critical role of acetyltransferase p300 in heart attack related cardiac pathologies, and thus acetyltransferase p300 is a potent target of epidrug development for therapy of cardiovascular pathologies (Figure 2).

## 4. Epigenetic Regulation of Aortic Valve Calcification by Acetyltransferase p300

Aortic valve calcification is one of most common cardiac disorders in aged patients (>60 years) or younger patients with kidney disease. Cardiac valve calcification is characterized by disruption of calcium homeostasis, abnormal calcium deposition, and abnormal extracellular matrix accumulation on the leaflets of the aortic valve that leads to blockage or obstruction of blood outflow from left ventricle to entire body. Aortic valve calcification is prevalent in elderly patients with hypertension, diabetes and high LDL cholesterol (Reviewed in [79,80]). Like many other cardiovascular pathologies, the initiation and progression of aortic valve calcification pathology is also epigenetically regulated by acetyltransferase p300. Li and colleagues [81] reported that porcine valvular interstitial cells grown in osteogenic media with TNF-α and high phosphate display increased expressions or levels of osteocalcin, alkaline phosphatase, acetylated-H3K9 compared to control cells. Importantly, pharmacological inhibition of acetyltransferase p300 with small molecule C646, reduces osteocalcin levels and blocks osteogenesis of porcine valvular interstitial cells [81]. Therefore, acetyltransferase p300 is a putative epidrug target for the treatment of aortic valve calcification. Furthermore, treatment of porcine aortic valve interstitial cells with high calcium/high phosphate causes increased calcium deposition and osteogenesis like aortic valve calcification phenotype [79]. Interestingly, acetyltransferase p300 inhibitor C646 prevents the high calcium/high phosphate induced acetylation of H3K14, H3K27, and H4K56 residues, calcineurin deposition and osteogenesis in valvular interstitial cells. These results further indicate that acetyltransferase p300 plays a significant role in aortic valve calcification through increased acetylation of specific histone lysine residues. In contrast, HDAC-inhibitor SAHA activates valvular interstitial cell calcification. Furthermore, the levels of H3 and H4 acetylation are significantly elevated in adenine and Vit D-induced calcified aortic valves in mice. Importantly, acetyltransferase p300 inhibitor C646 ameliorates adenine/VitD-induced aortic valve calcification through suppression of adenine/VitD-induced H3 and H4 acetylation [79]. Therefore, these studies clearly indicate that normalization of elevated p300 acetyltransferase activity using epidrug-like small molecule inhibitor is an ideal approach to controlling aortic valve calcification, an indicator of possible heart failure (Figure 2).

## 5. Perspective and Future Direction

The essential roles of acetyltransferase p300 in cellular senescence/aging (Figure 1), and related cardiovascular pathologies (Figure 2) through acetylation of histones and transcription factors of genes involved in regulation of cellular senescence/aging and cardiovascular pathologies are well documented in the literature by numerous cellular and animal studies. Therefore, acetyltransferase p300 is considered as a potent target of epidrug development for mitigation of the accelerated cellular aging processes and aging-related cardiovascular diseases. Although, several small molecule inhibitors targeting acetyltransferase p300 have been successfully used in different preclinical setups to ameliorate different cardiovascular pathologies, thus far none of these small molecule inhibitors has moved to clinical trial for the treatment of aging related cardiovascular disease. Therefore, it is desirable to develop new safe and potent epidrug targeting acetyltransferase p300 for the therapy of aging-related cardiovascular diseases. As physiological levels of acetyltransferase p300 activity is also involved in a wide variety of cellular and biological processes including cellular proliferation, adhesion, differentiation, muscle cell attachment, and myogenesis, complete inhibition of acetyltransferase p300 for long-term therapy of cardiovascular disease may cause detrimental effects like muscular dystrophy phenotype [82]. Therefore, normalization of acetyltransferase p300 activity under pathological milieus is crucial for more effective and safe treatment of cardiovascular disease.
